# 

**Published:** 1888-07-07

**Authors:** 


The population of the little county towns of England is stationary or retrogressive, while that of the cities is swelled out of all proportion. In 1861 there were already 172 dwellers in towns to 100 dwellers in rural districts; in 1871 the number had risen to 192; in 1881 it had reached 212, or somewhat more than two to one. London in this respect is typical. Its population now exceeds that of Paris, Berlin, Vienna, and St. Petersburg combined. It is larger than the aggregate population of our nineteen largest provincial towns. London is now adding to her enormous population at the rate of 70,000 souls a-year, and its dangerous plethora is swelled by the continuous influx of pauper foreigners, who almost from the week of their arrival begin to live on the poor-rates. —*Archdeacon Farrar.*

IT is important to observe that although hard physical work tends to shorten life, hard mental work has nothing like the same effect for evil. Indeed, the longest livers are those who do a large amount of intellectual work. With such regulations as it possesses, and the very great number of public commons surrounding it, London may continue to grow in size, but not in density of population, and will continue to be the healthiest large city in the world.—*J. A. Russell, M.B., F.R.S.E.*

PURE air is to the lungs the most important tonic, and we should see to it that we have sufficient of it in the rooms in which we live and work. Not only must the air-space be sufficient, but that air must be constantly renewed, if we are to live healthy lives. Nor can the importance of open air exercise be exaggerated. We should accustom ourselves and our children to be out in all weathers, and at all temperatures, unless there be some special reason to the contrary. Keep the skin healthy with regular cold bathing, and always wear wool next to it.

**Special Notice to Newsagents, Advertisers, and the Trade.**

**CHANGE OF OFFICE AND PUBLISHER.**

NOTICE is hereby given that the Office of THE HOSPITAL will henceforth be at 33, Old Jewry (Cheapside end), E.C., to which address all communications should be sent. Mr. A. Doig has been appointed Advertising Agent, and Mr. J. H. S. Hanning, Manager. All Cheques and Post Office Orders should be made payable to J. H. S. Hanning, 38, Old Jewry, E.C., crossed Lloyds, Barnetts, and Bosanquets Bank (Limited).

**To Secretaries and Matrons.**

THE Editor will be glad to have a copy of the Regulations, and also of every Report issued from the Press of Asylums, Hospitals, Convalescent and Nursing Institutions, as well as those of other Charities, so soon as they are published, and an early copy in duplicate of all appeals for funds. Notices of Annual and other Meetings, Festival Dinners, etc., will be welcomed. All such communications should be addressed to *The Editor, The Lodge, Porchester Square, London, W.* Any superintendent, secretary, matron, or other chief official who will regularly send such documents and information will be registered to receive free of cost a cover for binding THE HOSPITAL in half-yearly volumes on sending name and address to the publisher.

### Amusements with Profit for all Ages.

**Rules.**—All answers must be addressed to the Prize Editor, The Hospital, 38, Old Jewry, Cheapside, London, E.C., not later than the *first* post on Thursday next. The full name and address must in all cases be given, but a *nom de plume* may be added for publication. Only one side of the paper must be written on.

### FOR MEN AND WOMEN.

### First Quarterly Poetical Competition.

Commenced May 26th, 1888; ends August 25th, 1888.

This quarter a PRIZE of ONE GUINEA will be given to the competitor who gains the highest number of marks for original poems, on any subjects suitable for insertion in this paper. All contributions sent in will be credited according to merit, eighteen marks being the highest score for any one contribution. This competition to alternate weekly with the set acrostic competition.

### Acrostic Competition.

This quarter the original acrostic competition will be discontinued, and **A PRIZE of HALF-A-GUINEA will be given to the competitor who gains the highest score for solution of acrostics set. *One mark* only will be given to the competitors who solve all the lights of each acrostic.**

Exception will be made in the case of ***quotation acrostics***, when *two marks* will be given, ***one*** for the correct solution of all ***lights***, and *one* mark for the ***source*** of the quotations.

This week credit will be given for solution of

### No. 3.—Double Acrostic.

Not the true calm of well-based confidence,
But opiate stupor of our England's sense;
Haste, cut it short, ere yet 'tis called too late.
"If England to herself do prove but true,"
*This* need we urge: Then "nought shall make us one,"
Who faced aright the crisis of our fate.

1. Alive seems dead; awake, asleep;
   Entrancéd state enforced to keep
2. Suits the pure heart, but not the stalwart hand,
   When furious foes assault the fatherland.
3. Ill fares the mood which, ostrich-wise,
   E'en courts the danger it denies.
4. In a brief hour it sweeps, or so they say,
   Vast adamantine piles in dust away.
5. Up, one and all, who bear this name;
   Forfend your country's loss and shame.
6. Trust not this quack, writ large, whose glozing
   Would keep our nation to its ruin dozing.
7. 'Tis short or none if finals should go wrong;
   Then comes repentance, all too late and long.

### Answers.

No. 2.—Double Acrostic.

| | | |
|---|---|---|
| H | oo | P |
| U | n | A |
| M | ara | T |
| O | rpa | H |
| U | golin | O |
| R | assela | S |

Correct answers have been received from Mater, Dodo, Rowdy Corner, Ruffles, Alicia, Reldas, Ostrich, Lady Clara (2).

*Lady Clara.*—You are credited with two marks this week, your name having inadvertently been omitted from No 1.

*Jonah.*—Your word dissections received, but being past date cannot be credited.

### THE WORD COMPETITION.

Third Quarterly Competition commenced

May 5th, 1888; ends August 4th, 1888.

Three prizes of one guinea, 15*s.* and 10*s.* 6*d.* will be given each quarter to the three competitors who obtain the highest number of marks by making the largest number of words derived from the word or phrase set for anatomical dissection; that for this, the *ninth* week of the quarter, being

"Universal."

**Observe.**—Proper names, abbreviations, foreign words, words of less than four letters, and repetitions are barred; plurals, and past and present participles of verbs are allowed. Nuttall's *Standard* dictionary only to be used.

**N.B.**—All words sent in must be arranged ***alphabetically***, with correct total affixed.

### Results of Seventh Week.

*Emigration.*—Time (267), Patience (265), Lightowlers (254), Dodo (254), Reldas (253), Lauriston (251), Puff (246), Dollie (235), Sym (229), Oliver (216).

### THE CHILDREN'S CORNER.

PRIZES.

FOR MOST CORRECT ANSWERS TO PUZZLES.

This Commenced October 1st, 1887.

One Pound a Month.

A PRIZE OF THREE GUINEAS

to the Competitor who shall have sent in the largest number of correct or best answers during the twelve months.

### Puzzles.—First Week.

No. 1. Double Acrostic.—The initials give the name of a well-known author, the finals what he is best known as. 1. A kind of race. 2. A well-known dentifrice. 3. An opening piece of music. 4. The reverse of sadness.

No. 2. Riddle-me-ree.

My *first* is in hare, but not in game.
My *second* is in salmon, but not in same.
My *third* is in strife, but not in foe.
My *fourth* is in sorry, but not in woe.
My *fifth* is in field, but not in mow.

No. 3. Geographical Charade.

My *first* is a point, but I don't say of what.
My next we oft say when we've some. Who would not?
I would, if my *second* applied to my *third*.
My whole is a county. Now tell me the word.

No. 4. Enigma.

I produce both sound and melody.
Read me in another sense, and without
Me, neither noise nor melody would exist.

### Results of Third Week Puzzles.

No. 11. Logogriph.—One, sling, onion, live, ton. The traveller was Livingstone.

No. 12. Historical Mental Picture.—The queen was Elizabeth, the nobleman the Earl of Essex, and the politician Lord Burleigh.

No. 13. Charade.—Arrow Root=Arrowroot.

No. 14. Enigma.—A shadow.

*All right*—Gem, Gennaro, Alsie, Scrooge, Kensington, W. R., R. H., Thanet, Happy Eliza, Judy. *Three right*—A. C. K., Alsie, Plummy Fluff, Hercules.

*Condorcet.*—Kensington has been credited with five marks for first week's puzzles.

### Conditions.

I. Every paper sent in must be clearly written, and one side only must be used. All competitors must mark at the head of their papers the number of their competition.

II. Each paper must be signed by the author with his or her real name and address. A *nom de plume* may be added, if the writer does not desire to be referred to by us by his real name. In the case of all prizewinners, however, the real name and address will be published.

III. All communications relating to prize competitions should be addressed to "The Prize Editor, The Hospital, 38, Old Jewry, London, E.C." Competitors are requested not to include in letters, etc., to the Prize Editor communications on matters of other business. All letters must be fully prepaid.

IV. The Prize Editor of The Hospital is the sole judge of the competitions, and his decision is in all cases final and without appeal.

V. The Prize Editor reserves the right to publish any paper sent in, whether it receives a prize or not. He does not hold himself responsible for any MSS. sent, nor can he undertake to return rejected competitions.

VI.—All children resident at school are requested to forward their *home* address in addition to the school one, when entering for the "Children's Corner" Competitions.

**Notice to all Correspondents.**—N.B.—All letters referring to this page, which do not arrive at 38, Old Jewry, Cheapside, London, E.C., by the *first post on Thursdays*, and are not addressed PRIZE EDITOR, will in future be disqualified and disregarded.

No one will be permitted to win the Monthly Prizes twice in any one year, the Annual Prize being offered especially to prevent this.

